# Delivery of Aerosolized Liposomal Amikacin as a Novel Approach for the Treatment of Nontuberculous Mycobacteria in an Experimental Model of Pulmonary Infection

**DOI:** 10.1371/journal.pone.0108703

**Published:** 2014-09-29

**Authors:** Sasha J. Rose, Mary E. Neville, Renu Gupta, Luiz E. Bermudez

**Affiliations:** 1 Department of Biomedical Sciences, Oregon State University, Corvallis, Oregon, United States of America; 2 Department of Microbiology, Oregon State University, Corvallis, Oregon, United States of America; 3 Insmed Incorporated, Monmouth Junction, New Jersey, United States of America; University of Lausanne, Switzerland

## Abstract

Pulmonary infections caused by nontuberculous mycobacteria (NTM) are an increasing problem in individuals with chronic lung conditions and current therapies are lacking. We investigated the activity of liposomal amikacin for inhalation (LAI) against NTM *in vitro* as well as in a murine model of respiratory infection. Macrophage monolayers were infected with three strains of *Mycobacterium avium*, two strains of *Mycobacterium abscessus*, and exposed to LAI or free amikacin for 4 days before enumerating bacterial survival. Respiratory infection was established in mice by intranasal inoculation with *M. avium* and allowing three weeks for the infection to progress. Three different regimens of inhaled LAI were compared to inhaled saline and parenterally administered free amikacin over a 28 day period. Bacteria recovered from the mice were analyzed for acquired resistance to amikacin. *In vitro*, liposomal amikacin for inhalation was more effective than free amikacin in eliminating both intracellular *M. avium* and *M. abscessus*. *In vivo*, inhaled LAI demonstrated similar effectiveness to a ∼25% higher total dose of parenterally administered amikacin at reducing *M. avium* in the lungs when compared to inhaled saline. Additionally, there was no acquired resistance to amikacin observed after the treatment regimen. The data suggest that LAI has the potential to be an effective therapy against NTM respiratory infections in humans.

## Introduction

Pulmonary infections caused by nontuberculous mycobacteria (NTM), specifically *Mycobacterium avium* subsp. *hominissuis* (MAH) and *Mycobacterium abscessus* (Ma), are increasing in incidence. MAH infection of the airways (and likely Ma as well), has been associated with the formation of biofilm [1], [2], [3]. Disease in patients with underlying lung pathology, such as cystic fibrosis and bronchiectasis, is especially difficult to treat, because the basic lung condition significantly affects the ability of the host to clear the pathogens. Treatment with macrolides, such as clarithromycin or azithromycin, in combination with ethambutol and rifampin is typically effective against MAH pulmonary infections [4], [5]. Streptomycin and amikacin can also be supplemented with the MAH regimen unless tolerability and toxicity become an issue. Macrolides are generally ineffective against Ma because it possesses *erm*, an inducible macrolide-resistance gene [6], [7]. Ma pulmonary infection is typically treated with cefoxitime, imipenem, and amikacin. Newer drugs, such as tigecycline and linezolid, show good *in vitro* effectiveness against Ma and are used in some instances [4]. The described treatment regimens for MAH and Ma are very long, many of the drugs require parenteral administration, and bacterial resistance to these drugs is a concern. Therefore, the limited activity of regimens and the lack of alternative therapeutic options make the treatment of these infections challenging.

NTM reside and multiply in both macrophages in the airway submucosa as well as in alveolar macrophages, which requires that compounds achieve bacteriostatic or bactericidal activity intracellularly [8], [9]. Being able to deliver high concentrations of a compound intracellularly might be crucial for the ability to effectively treat the infection [4], [10]. Liposomes are an attractive drug delivery system that can allow drugs with typically poor membrane penetration (e.g. aminoglycosides) to accomplish these objective.

The use of aminoglycosides encapsulated in liposomes has been shown to be effective as anti-*M. avium* both in macrophages *in vitro* and in mice when delivered intravenously [11]. Liposomal encapsulation also permits sustained release of high concentrations of the aminoglycoside, which translates into less frequent administration. Amikacin is particularly active against both MAH and Ma, but the narrow toxicity index for the compound and the need for parenteral administration make its current application limited [4].

A new experimental liposome preparation, liposomal amikacin for inhalation (LAI), composed of dipalmitoylphosphatidilcholine (DPPC) and cholesterol containing encapsulated amikacin, has been developed for aerosol delivery for respiratory infection. Inhalation of LAI to the respiratory tract lowers the potential for ototoxicity and nephrotoxicity compared to systemic administration of amikacin and maintains higher concentration of drug in the lung and at the site of infection [12]. LAI was effective for the treatment of *Pseudomonas aeruginosa* infection in a model of cystic fibrosis in rats and was shown to penetrate the *P. aeruginosa* biofilm [12]. Recently, a phase II study using LAI for the treatment of *P. aeruginosa* in cystic fibrosis patients was reported, demonstrating the short-term efficacy, safety, and tolerability of the drug [13]. In this current study, we established the efficacy of LAI against both intracellular MAH and Ma *in vitro* as well as in an experimental murine MAH respiratory infection.

## Materials and Methods

### Ethical Statement

All the performed experiments were carried out according to the guidelines for animal ethics. All the experiments were reviewed and approved by the IACUC committee of Oregon State University (ACUP # 4257).

### Bacteria

*M. avium* subsp. *hominissuis* strains MAH 104 and MAH A5 are blood isolates from patients with AIDS. MAH 3388 is a lung isolate from a patient with chronic pulmonary disease (kindly provided by Dr. Richard Wallace, University of Texas Health Science Center at Tyler, Tyler TX). *M. abscessus* subsp. *bolletii* Ma 26 and Ma 36 lung isolates were kindly provided by Dr. Steve Holland (National Institutes of Health). Each of the isolates was identified as MAH and Ma by using a commercially available DNA probe (Gen-Probe, San Diego CA). Ma 26 and 36 were further identified as subsp. *bolletii* by amplification and direct sequencing of the *erm*(41), *rpoB*, and *23s* genes by standard procedures and the primers used were sourced from Teng *et al.* [14]. MAH 104 and A5 have been shown to be virulent in mice, but he other strains have not been tested in experimental models to date. MAH and Ma were cultured on Middlebrook 7H10 agar (Hardy Diagnostics, Santa Maria, CA), supplemented with oleic acid, albumin, dextrose, and catalase (OADC; Hardy Diagnostics, Santa Maria, CA) for 10 and 4 days, respectively, at 37°C. Only pure clones (transparent colonies) were used in the study.

### Antimicrobial Agents

Amikacin sulfate was purchased from Hospira, Inc. (Lake Forest, IL) and LAI (Lot #232-012-015 for *in vitro* experiments, Lot #3-NFF-0184 for *in vivo* experiments) was provided by Insmed Incorporated (Monmouth Junction, NJ). LAI is a liquid dispersion consisting of 70 mg/ml amikacin encapsulated in liposomes prepared from dipalmitoylphosphatidilcholine (DPPC) and cholesterol (2∶1 mole ratio at a total lipid concentration of 40 mg/ml) in 1.5% NaCl. Placebo liposomes were made of DPPC and cholesterol at the same mole ratio and concentrations as LAI but contained no amikacin.

### Macrophage Test System

The source of macrophages was the THP-1 human monocyte cell line (ATCC, Manassas, VA) cultured in RPMI-1640 medium (Gibco, Chicago, IL) supplemented with 5% fetal bovine serum (Gemini, Sacramento, CA) and 2 mM of L-glutamine. THP-1 cells were maintained at 37°C in an atmosphere of 5% CO_2_. Assays were performed as previously described, with minor modifications [15]. Monocytes were grown to 5×10^6^ cells per ml, washed and resuspended to a concentration of 1×10^6^ cells per ml, and 1 ml was seeded into a 24 well tissue culture plate (Costar, Cambridge, MA). Monolayers were then treated with 0.5 µg of phorbol myristate acetate per ml for 24 hours to stimulate the maturation of the monocytes.

Bacteria were prepared for infection by resuspension in Hank's buffered salt solution (HBSS) to concentrations of 3×10^8^ CFU/ml by comparison with a McFarland #1 turbidity standard. Prior to the infection of macrophage monolayers, the suspension was vortex agitated for 2 minutes and passed through a 23-gauge needle ten times to disperse clumps. Microscopic observation confirmed the dispersion of the inoculum. Suspensions were serially diluted and plated onto 7H10 agar to confirm the concentration of the inoculum.

The monolayers were infected with MAH or Ma at a multiplicity of infection of 10∶1. After 1 hour of infection, the extracellular bacteria were removed with 3 HBSS washes, and the intracellular infection was allowed to incubate for 24 hours. Following the establishment of the infection baseline, the addition of LAI or controls was performed once daily for 4 days. Lysis of THP-1 cells was carried out with a 10 minute incubation in 0.1% Triton X-100 in sterile H_2_O followed by mixing, diluting, and plating onto 7H10 agar plates for CFU enumeration.

### Mice

Six week-old female C57BL/6 mice were purchased from The Jackson Laboratory (Bar Harbor, ME) and delivered to the Laboratory Animal Research Center at Oregon State University, where they were acclimated for 7 days prior to experimentation.

### Experimental Respiratory Infection of Mice

Mice were infected intranasally with 2.25×10^7^ CFU of MAH strain 104. To minimize sneezing or aspirating, each mouse was lightly sedated with brief exposure to isoflorane prior to inoculation. Infection was allowed to establish for three weeks, where at that point 12 mice were euthanized to determine the pre-treatment infection burden. Five groups of 12 mice were used for treatment, including: 1 hour of saline inhalation every day for 28 days; 1 hour of LAI inhalation every day for 28 days; 2 hours of LAI inhalation every day for 14 days, then 14 days of no treatment; 2 hours of LAI inhalation every other day for 28 days; IP injected free amikacin IP administered once every day for 28 days. A 12 port Jaeger-NYU nose-only directed flow inhalation system (CH Technologies, Westwood, NJ) was used in conjunction with a Devilbiss 8650d compressor (Devilbiss, Jackson, TN) and LC Star nebulizers (Pari, Midlothian, VA), connected by flexible tubing (supplied with the nebulizers). The mice are fully conscious and not anesthetized while in the plastic restrainers for the duration of treatment. Five (5) ml of test article (either 1.5% NaCl or LAI) was placed inside a nebulizer and the system ran for 20 minutes at 30 psi. This was performed 3 times consecutively to achieve an inhalation time of 1 hour and 6 times consecutively to achieve an inhalation time of 2 hours. The nebulizers were weighed before and after every 20 minute cycle to assure consistent nebulization. The actual dose of delivered LAI into the plastic restrainers was determined multiple times throughout the 28 day treatment regimen, following a previously published protocol [16]. Amikacin sulfate was diluted in sterile HBSS to 100 mg/kg and administered intraperitoneally (IP) using a 28 gauge needle and restraining the non-anesthetized animal by a scruff grip.

### Extraction and Enumeration of Bacteria from Mice

Necropsies were performed and the lungs (containing bronchus and 0.5 cm of trachea) were aseptically excised and homogenized using a Bullet Blender (Next Advance, Averill Park, NY) following the manufacturer protocol for bacterial isolation from host tissue. Homogenized samples were then serially diluted and plated onto 7H10 agar plates for CFU enumeration.

### Measuring Amikacin Resistance of Bacteria Recovered from Mice

Two methods were employed to assess resistance of bacteria to amikacin. First, homogenate was serially diluted and plated onto 7H10 to allow individual colonies to grow. Individual colonies from three mice of each group were transferred into 7H9 broth to 3×10^8^ CFU per ml using visual turbidity. Individual colonies from lab-maintained MAH 104 were used to assess the baseline minimum inhibitory concentration (MIC) of the strain. Final solutions of 3×10^5^ CFU per ml in 0.5 through 64 µg/ml of amikacin, in 7H9 broth, were shaken at 37°C for 5 days. At this point, MICs were determined by assessing visual turbidity. Second, sample homogenates from groups all treatment groups were plated directly onto 7H10 agar with no antibiotic, 7H10 containing 32 µg/ml of amikacin, and 7H10 containing 64 µg/ml of amikacin. Plates were incubated at 37°C for 14 days and CFU data was compared between the antibiotic and no antibiotic plates.

### Statistical Analysis

Pairwise comparisons made for the *in vitro* experiments were determined by the Mann-Whitney non-parametric test. Comparisons between multiple groups were conducted with ANOVA. Graph Pad Prism (La Jolla, CA) was used for statistical analyses and graphical outputs.

## Results

### LAI is More Effective than Free Amikacin Against Intracellular Mycobacteria *In Vitro*

**Figure 1:**
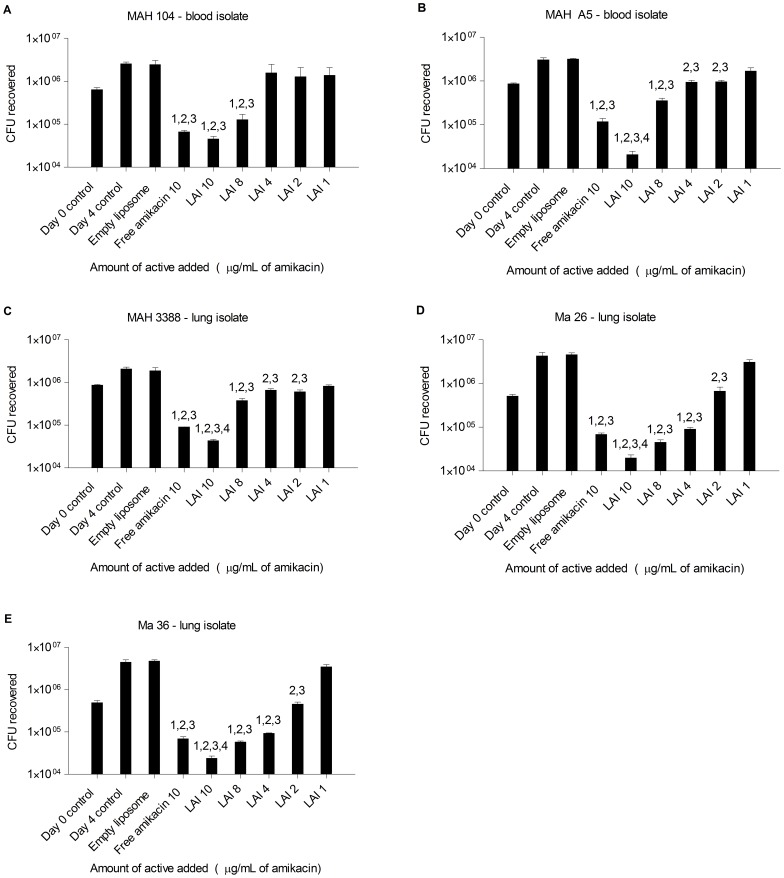
Efficacy of LAI against intracellular mycobacteria *in vitro*. LAI, a liposome-encapsulated amikacin compound, was tested against intracellular *M. avium* subsp. *hominissuis* (MAH) and *M. abscessus* (Ma) *in vitro*. THP-1 human cells (that were first differentiated and adhered with PMA) were infected with a 10∶1 MOI of MAH strains 104 and A5, which are blood isolates (A and B, respectfully), MAH 3388, a lung isolate (C), and Ma strains 26 and 36, which are also lung isolates (D and E, respectfully) for 1 hour. Extracellular bacteria were removed and cells containing internalized bacteria were incubated at 37°C for 24 hours. Wells were either plated for CFU (day 0 control) or treated daily with HBSS (day 4 control), empty liposome, 10 µg/ml amikacin sulfate, or between 10 and 1 µg/ml of LAI for 4 days. Cells were disrupted, diluted, and plated to obtain CFUs of surviving bacteria. Bars represent means of CFU counts and error bars represent standard deviation. Statistical comparisons: 1 = p<0.05 compared to day 0 control; 2 = p<0.05 compared to day 4 control; 3 = p<0.05 compared to empty liposome; 4 = p<0.05 compared to free amikacin.

Previous work has demonstrated the effectiveness of LAI against extracellular *P. aeruginosa* in a rat model of respiratory infection [12]. The aim of this study was to assess the efficacy of LAI against intracellular mycobacteria. It has been found that fluorescently labeled LAI is internalized by macrophages, via co-localization of it with the intracellular environment (unpublished data), which introduces the possibility of testing it against intracellular pathogens. We first investigated the effectiveness of LAI against *Mycobacterium avium* subsp. *hominissuis* (MAH) and *Mycobacterium abscessus* (Ma) *in vitro*. Monolayers of adherent THP-1 macrophages were infected with approximately 1×10^7^ MAH for 1 hour. The extracellular (non-phagocytized) bacteria were removed by multiple washes, and the macrophages containing intracellular bacteria were incubated at 37°C for 24 hours and then treated with either compounds or controls. Free amikacin was used at 10 µg/ml and LAI was used at 1, 2, 4, 8, and 10 µg/ml of amikacin. MAH strains 104 (blood isolate), A5 (blood isolate; biofilm overproducer), and 3388 (lung isolate), were significantly inhibited by free amikacin and LAI, compared to the day 4 control and empty liposome treatment (Figure 1 A–C). At the concentration of 10 µg/ml, LAI treatment of MAH A5 and MAH 3388 was associated with significantly more killing compared with 10 µg/ml of free amikacin (Figure 1 B and C, respectively).

Macrophages infected with the strains Ma 36 and Ma 24 were treated with both amikacin and LAI leading to the killing of the intracellular Ma (Figure 1 D and E). LAI at 10 µg/ml was significantly more effective than free amikacin for both strains. Reductions in Ma CFUs appeared to show a linear dose dependence for LAI. Collectively, this demonstrates the effectiveness of LAI *in vitro* against intracellular NTM infection of macrophages.

### LAI is comparable to Parenterally Administered Free Amikacin against Intracellular Mycobacteria in a Murine Respiratory Infection

Due to the effectiveness of LAI against intracellular mycobacteria *in vitro*, an established murine model was used to evaluate the efficacy of LAI for treatment of an experimental MAH respiratory infection [3], [17]. Mice were inoculated intranasally with MAH strain 104 and infection was allowed to establish for three weeks. Three different inhaled treatment regimens of LAI were compared with inhaled saline and 100 mg/kg/day free amikacin by IP injection. The three LAI treatment groups included 76 mg/kg/day (1 hour) for 28 consecutive days, 152 mg/kg/day (2 hours) for 14 consecutive days (and then 14 days of no treatment), and 152 mg/kg/day (2 hours) every other day over 28 days. Importantly, all three groups received the same cumulative amount of LAI by the end of the 28 day period. LAI was delivered at a lower daily dose (76 mg/kg/day) compared to injected soluble amikacin (100 mg/kg/day) based on data collected throughout the treatment regimen that determined the delivered dose. After 28 days of therapy, all mice were euthanized and lungs were processed for CFU.

**Figure 2:**
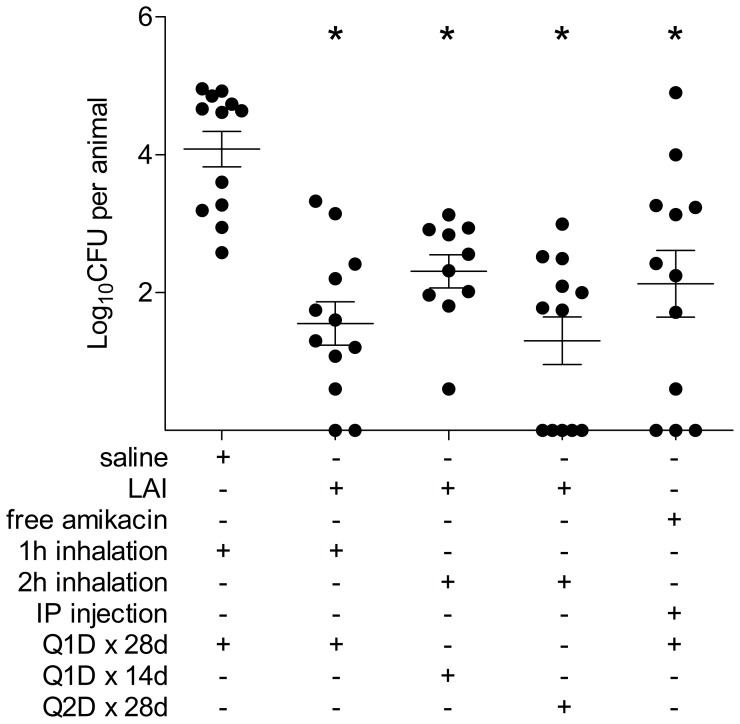
Efficacy of inhaled LAI compared to parenterally administered free amikacin in murine respiratory infection. Six groups of 12 mice were intranasally infected with MAH 104 and a respiratory infection was allowed to establish for three weeks. At this point, an initial group of 12 mice were euthanized to determine the pre-treatment burden of MAH 104 in the respiratory tract. The other 5 groups received either saline, LAI, or amikacin sulfate by various delivery routes and treatment regimens, as listed in the figure. Twenty-eight days after the initiation of treatment, all mice were euthanized and lungs were processed to determine the post-treatment MAH 104 burden. Dots resting on the x-axis are animals that had no culturable MAH in their lungs. Abbreviations: QID×28d = treatment every day for 28d; Q1D×14d = treatment every other day for 14d, then no treatment for 14d; Q2D×28d = treatment every other day for 28d. Horizontal lines represent the mean and SEM. Statistical comparisons: * = p<0.05 compared with inhaled saline control.

All amikacin treatment groups significantly reduced the MAH burden in the mice, when compared with the inhaled saline control group (Figure 2). Furthermore, the groups that received one hour daily LAI inhalation and two hours every other day cleared more MAH than the group that received IP injected amikacin, despite the fact that these animals received 32% less amikacin over the course of the study (p not significant). The group that received two hours of LAI inhalation every other day also had more animals without culturable MAH than the IP injected amikacin group (42% and 25% of animals, respectively; Figure 2).

Bacteria recovered from lung homogenates were assessed for acquired resistance to amikacin, using two methods. First, homogenate from saline and LAI treated mice were plated onto 7H10 agar without antibiotic, and 7H10 containing 32 or 64 µg/ml of amikacin. No bacteria grew at either amikacin concentration for any of the homogenates tested (Table 1). Second, a broth dilution method was used to determine the actual minimum inhibitory concentration of MAH from various treatment groups to amikacin, which also yielded no differences from the saline treated and laboratory-maintained bacteria (Table 2). Both of these experiments indicate that resistance to amikacin was not acquired by MAH during the 28 days of therapy. Taken together, this data suggests that inhaled LAI treatment is effective against MAH infection of the airways in mice, and resistance to amikacin was not observed after the treatment regimen.

**Table 1:** Amikacin resistance testing on bacteria grown from lung homogenate after treatment regimen.

Source of bacteria		Bacterial growth (Y/N)		
Treatment regimen	# of mice sampled	0 ug/ml	32 ug/ml	64 ug/ml
Saline 1h Q1D×28d	5	Y	N	N
LAI 1h Q1D×28d	5	Y	N	N
LAI 2h Q1D×14d	3	Y	N	N
LAI 2h Q2D×28d	1	Y	N	N

**Table 2:** Amikacin minimum inhibitory concentration determination on colonies isolated from lung homogenate after treatment regimen.

Treatment regimen	# of mice sampled	# of colonies tested*	MIC (µg/ml)
Saline 1h Q1D×28d	2	6	4
LAI 1h Q1Dv28d	2	6	4
LAI 2h Q1D×14d	2	6	4
LAI 2h Q2D×28d	2	6	4
IP amikacin Q1D×28d	2	6	4
lab-maintained control†	N/A	3	4

* three (3) colonies were isolated and tested from each mouse lung homogenate.

† lab-maintained MAH 104 (not recovered from infected mice) was used as a baseline.

## Discussion

Nontuberculous mycobacteria (NTM) are a cause of serious infection in patients with chronic pulmonary conditions. These infections progress slowly and in many cases have unsatisfactory response to antibiotic therapy [4]. Although MAH infections appear to respond to therapy initially, both the course of MAH and Ma infections are known to progress in despite of antimicrobial therapy. Therefore, new forms of therapy with limited toxicity are urgently needed. Inhaled preparations of aminoglycosides, that are active in the lung but have limited distribution in other tissues and that are associated with less toxicity than the free drug, are potentially a useful approach for new therapy.

In this study, liposomal amikacin for inhalation (LAI) showed significant activity against both intracellular MAH and Ma in a macrophage model *in vitro*. Notably, LAI was superior to comparable concentrations of free amikacin. The dynamic conditions in the lungs should favor LAI because of its deposition of higher local concentrations of amikacin. In fact, LAI reduced and eradicated *P. aeruginosa* in a rat lung model more effectively than inhaled free amikacin [12]. However, this is the first time that the activity of LAI has been evaluated in an established *in vivo* system against an intracellular pathogen versus parenterally injected amikacin. In the present murine model, both inhaled LAI and parenteral administration of amikacin were very effective in reducing the CFU of MAH in the lungs of mice compared to inhaled saline. In a recent report describing a new experimental murine *M. tuberculosis* model for testing aerosolized drugs [18], the authors demonstrated that aerosolized amikacin was comparable to subcutaneously injected amikacin at reducing *M. tuberculosis* in the lungs, even with a lower dose used and less frequent administration. This data agrees with our results showing the efficacy of LAI against MAH in mice. Inhaled dry powder containing antibiotic has also shown promise for the treatment of *M. tuberculosis* infection [19], [20], and has even been recently evaluated in a phase I study in healthy individuals [21]. Collectively, this recent work indicates that topical treatment of pulmonary mycobacterial infections could be feasible as a future treatment approach.

The *in vivo* effectiveness of LAI was comparable to injected free amikacin, even though a smaller dose of LAI was delivered. Although there was not a statistically significant difference in CFU reduction between the free amikacin group and two of the three LAI groups (one hour inhalation every day for 28 days and two hours inhalation every other day for 28 days), the mean CFU recovered in these two experimental groups were both smaller than the group treated with free amikacin, despite receiving 32% less drug. The data suggests that LAI might be more effective than the free amikacin in reducing MAH, which concurs with the observation *in vitro*.

It is well known that liposomal preparations achieve a greater intracellular concentration in phagocytes than free compounds [22]. It is more evident in the case of aminoglycosides, which have limited ability to cross membranes of mammalian cells [23], therefore only achieving a small fraction of the extracellular concentration intracellularly. Liposomal preparations can efficiently penetrate cell membranes, but the ability to cross and concentrate within phagosomes can vary with the preparation. Liposomal amikacin and gentamicin preparations have been evaluated against MAH and have shown to be as active or more active than the free drug, both in a macrophage system, as well as in mice when delivered intravenously [11]. A possible advantage of achieving high concentrations of a compound at the site of infection, beyond the greater anti-bacterial activity, is the decreased chance for the development of antibiotic resistance. In this current study, development of resistance to amikacin was not observed in bacteria recovered from both mice treated with LAI or free amikacin (Tables 1 and 2).

LAI was generally well tolerated in mice infected with NTM; however two animals were preemptively euthanized, following IACUC guidelines, due to adverse stress and subsequent weight loss (greater than 20% reduction from starting weight). These animals were in the group that received two hours of LAI daily for 14 days, and then no treatment for 14 days, even though they were euthanized before day 14. Both animals displayed abnormal posture, rough hair coat, reduced mobility, decreased alertness, and labored breathing. The attending veterinarian determined that these symptoms were a result of the weight loss. There did not appear to be any gross pathology upon necropsy. Even though they were not included in the data presented in this study, the CFU in the lung of one of these animals was obtained and was at a level consistent with the other animals in the group, ruling out that an adverse response to infection was the source of the weight loss. All of the other mice receiving LAI in all three LAI treatment regimens actually gained weight over the course of the study and had a higher average weight than the inhaled saline group at the end of the study, suggesting that LAI was not the cause of the weight loss either. Thus the stress of two hours of daily confinement in the inhalation chamber may have led to an eating disorder in these two animals, which has been seen in mice that are merely restrained and not treated with any test articles [24].

In summary, this study demonstrated that LAI is active against MAH and Ma *in vitro* at concentrations easily achieved in the airways and respiratory mucosa. Additionally, a lower dose of LAI administered topically via inhalation was comparable to parenterally administered amikacin in the treatment of MAH lung infection in mice, with no resistance to amikacin observed in bacteria recovered after the various treatment regimens. These data suggest that LAI be further investigated as a new treatment option for NTM respiratory infection. Currently, a phase 2 clinical trial with LAI for the treatment of patients with NTM infection is underway in the US, including at the National Institutes of Health and Infectious Diseases (ClinicalTrials.gov Identifier: NCT01315236).
